# Bio-invasive ascidians in Ireland: A threat for the shellfish industry but also a source of high added value products

**DOI:** 10.1080/21655979.2017.1392421

**Published:** 2017-12-01

**Authors:** Satheesh Kumar Palanisamy, Olivier P. Thomas, Grace P. McCormack

**Affiliations:** a Department of Zoology, School of Natural Science, Ryan Institute, National University of Ireland, Galway, Ireland; b School of Chemistry, Marine Biodiscovery, National University of Ireland, Galway, Ireland

**Keywords:** Aquaculture, biodiversity, biofouling, invasion, shellfish industry, ascidians

## Abstract

In October 2016, a rapid assessment survey of ascidian species was conducted in shellfish farms at Killary Fjord, in the west of Ireland. Two non-indigenous solitary ascidians *Ascidiella aspersa* and *Corella eumoyta* were recorded for the first time in shellfish farms at this location. Both invasive ascidians have the potential to greatly reduce mussel production in Killary Fjord by competing with shellfish for food and habitat. Their high abundance also causes an increase in maintenance costs leading to economic losses for aquaculture farmers. Prompted by our finding of two invasive ascidians in Killary Fjord, we provide a brief review of the ecological role of ascidians and the potential of harnessing biomass from such invasive species for the production of high added value marine natural products.

## Introduction

Ascidians, sea squirts or tunicates are benthic marine invertebrates present throughout the marine environment from the intertidal to the deep-sea. The high invasive potential of some marine species of this group represents a threat to marine biodiversity worldwide. Here we report the impact of non-indigenous ascidians (NIA) in Killary Fjord, Ireland (Latitude: 53° 36′ 59.99″ N and Longitude: −9° 47′ 59.99″ W). Killary Fjord, is located on the west coast of Ireland being 9.9 miles long with a highest depth of 45 m. It is the principal region for the aquaculture of the blue mussel *Mytilus edulis* in Ireland. Bivalves are cultivated in shallow waters using longlines and they are considered the finest and most highly demanded farmed mussels in Ireland and other European countries.^1^ Mussel farmers take advantage of the wild mussel population by collecting mussel seed for growing in Killary Fjord and in other parts of Ireland. However, some of the most promising sites for seed collection tend to be associated with invasive ascidian species.^2^

Ascidians are the most diverse class of the subphylum Tunicata. They comprise approximately 3,000 described species found in all marine habitats from shallow water to the deep sea, showing a large variation in form and colour.^3^ A review of the literature indicates that more than 65 ascidian species are known from Irish waters, six of them being considered as invasive like *Ascidiella aspersa, Didemnum vexillum, Styela clava, Botrilloides violaceous, Corella eumoyata* and *Perphora japonica*.^4^ Several modes of introduction of NIA are possible into the region of Killary Fjord. The first is through ship ballast water, as the ascidian larvae are able to postpone settlement and survive for several days.^5^ Ascidians also represent one of the main biofouling species, particularly on ship hulls.^6^ These organisms can colonise all types of hard substrate both natural and artificial, especially in the environment characterised by low diversity fauna such as coastal lakes, lagoons, harbours and shellfish farms. The aim of this review is to report on the distribution of invasive ascidians in Killary Fjord, assess their environmental impact, the socio-economic implication and the potential in the discovery of marine natural products.

### Impact of ascidians on shellfish farming

**Figure 1.**
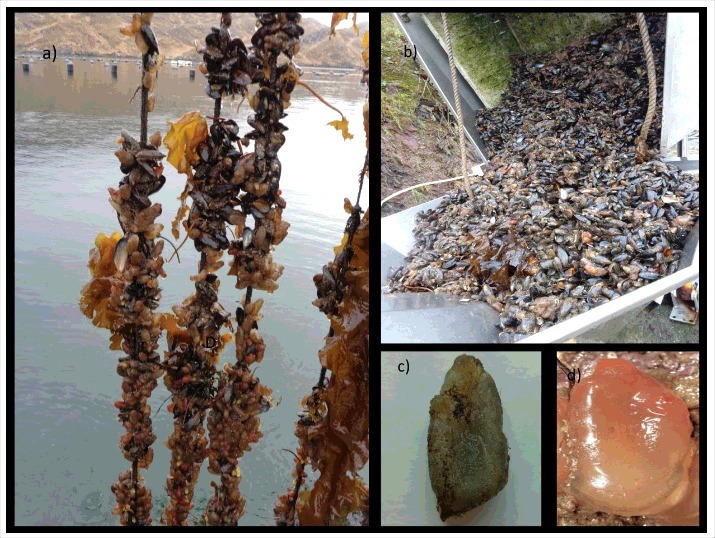
Invasive marine ascidians on blue mussels, Killary Fjord, Ireland. (a): Invasive ascidian communities attached to cultured mussels and biomass of cultured mussels is not even in longlines; (b) mussels with ascidians for machine cleaning and processing; (c) *Ascidiella aspersa; (d) Corella eumoyata*.

In 2013 mussel farmers in Killary Fjord identified some ascidian species as biofoulers of the shellfish lines and assumed they were not serious pests (Simon, Killary Fjords Shellfish Farm personal communication). From 2014 to 2016, the ascidian population increased massively expanding in Killary Fjord and other locations in Galway Bay. We subsequently recorded extensive biofouling of invasive solitary ascidians *A. aspersa*, and *C. eumoyata* along with other ascidians like *A. mentula, A. scabra* and *Ciona* sp (Fig. 1) at depths ranging from 3 to 15 m. The most common ascidians were the invasive species *A. aspersa* and *C. eumoyata*, both of which were recorded for the first time in Killary Fjords, and thus form new locality records. In previous studies, Minchin and co-workers^4,7^ reported the first appearance of non-indigenous ascidian species at various sites in Irish waters including *D. vexillum, S. clava, B. violaceous, C. eumoyata* and *P. japonica* while the biology of *A. aspersa* and *C. eumoyata* was reported^8,9,10^ *A. aspersa* is reported to tolerate wide seasonal fluctuations of oceanic parameter changes and is more adaptable to climate change and shifting geographic ranges. The spawning of this species was noticed throughout the year, but mature development was observed only in autumn.^11^ Remarkably, depth did not impact the reproductive cycle of *A. aspersa*. However, earlier gonad maturation and spawning were observed in individuals collected in deeper waters compared to shallow water depth.^8^

Ojaveer and co-workers reported the major issues caused by marine invasive species in European waters.^12^ Invasive and native tunicates are of economic concern for shellfish farmers because they overgrow shellfish and pollute the long line gear, thereby adding weight and restricting water exchange and nutrients.^2^ Ascidians are benthic filter feeders, and can lead to a decrease in the shellfish production (both bivalves and gastropods) due to competition for food.^2^ The first evidence of PSP toxins saxitoxin and gonyautoxins were reported in *Microcosmus vulgaris* harvested from the farmed mussels (*Mytilus galloprovincialis*) at west coast of Istria Peninsula, Croatia.^13^ NIA species have caused serious impacts on aquaculture at Killary by smothering the stocks, making aquaculture production labour intensive, decreasing the overall production, reducing the size of mussels and causing loss of income for shellfish farmers.

Dense populations of the invasive ascidians *C. intestinalis, A. aspersa, S. clava, C. eumoyata, D. vexillum* and other species were frequently found on aquaculture structures and hard substrates more widely in the nearby marine environment of Killary Fjord suggesting that the invasive species significantly contribute to the fouling community and may also have become the dominating biofouling organisms in the region.^13^ Recently, Ardura *et al.* reported that the abundance of NIA is lower in coastal marine protected areas of Canada and French Polynesia.^15^ In Irish waters, the introduction of invasive ascidians is also reported to be lower in marine protected areas compared to the coastal regions extensively affected by human activities e.g. Cork and Dublin harbour;^15^ Yacht clubs in Cork, Dublin,^4^ Howth fishing harbour, and aquaculture practice like in Killary Fjords, Galway. Previous reports of NIA distribution in Irish waters emphasized the importance of marine protected areas for marine biodiversity conservation and suggested that marine protected zones would confer efficient protection against NIA introduction.^4^ However, the lack of taxonomic expertise in the field of tunicate taxonomy, insufficient information on the distribution and origin of species and vectors of NIA species reported in the present study suggests that further research in this area is necessary.

### Commercial biocides against ascidian larval settlement

The prevention method used to deter marine biofouling organisms may vary depending on the species and the age of the shellfish being cultured. Removing the ascidians manually requires additional labour by shellfish farmers, leading to increased maintenance costs. The following techniques have been effectively utilised for the removal/control of marine biofoulers on shellfish; the application of lime,^16^ dilute bleach, vinegar, plastic wrapping and exposure to air^17^ and using biocides.^18^ All such practices can account for up to 25 –30% of the operational cost of shellfish farmers.

The commercially important anti-fouling biocides Irgarol-1051, Sea-Nine 211 and Chlorothalonil significantly inhibit embryonic development in *C. intestinalis*.^18^ The biocide, Chlorothalonil showed maximum toxicity against embryonic stages of *C. inetestinalis* (at concentration EC_50_ 33 µg/L), and following that, Sea-Nine 211 (EC_50_ 105 µg/L), Irgarol-1051 (EC_50_ 2115 µg/L) and zinc pyrithione (Zpt) (EC_50_ 108 nM)^17^ also inhibited larval settlement. Over all, Chlorothalonil was revealed 70 times more toxic than Irgarol-1051 and was three times more toxic than Sea-Nine 211.^19^

Furthermore, Sea-Nine 211 and Chlorothalonil showed the reduction of haemocyte functionality of colonial ascidian *Botryllus schlosseri* at concentration 1 and 10 µM, which is comparable to that of Tributyltin (TBT).^20^ Similarly, the other biocides Diuron and TCMS pyridine showed sublethal activity on haemocytes of *B. schlosseri* by changing the cytoskeleton and cell morphology and inducing the impairment of DNA.^21^ Additionally, the synthetic catamine and medetomidine induced metamorphosis and reduced the larval settlement of ascidian *S. clava* at concentration 20 mg/L. Further, medetomidine increased the rate of larval immobility with EC_50_ values of 3.8 mg/L in 2 h.^22^ The use of commercial anti-fouling painting maintains the colonization of the ascidian *Styela clava* on boat hulls to a minimum, even after 12 weeks in seawater.^22^ The results of the previous studies suggest that these biocides can control biofouling communities and non-target benthic organisms. However, such biocides have to be used with caution as ecotoxicological studies on other micro and macro organisms of the ecosystem are still needed. Further investigation and long-term monitoring data are required to evaluate the bioaccumulation of synthetic biocides by benthic organisms before their use against NIA species in finfish /shellfish aquaculture systems.

### Anti-fouling marine natural products

Settlement and metamorphosis are subsequent critical events in the life cycle of sessile marine organisms to obtain a suitable habitat for food and reproduction. Although diverse physical factors have been reported that influence larval settlement and metamorphosis, metabolites derived from the adults, from prey, or from bacterial films are believed to be more important.^23^ In addition to purely synthetic compounds, a number of natural products have been reported from marine organisms such as sponges, ascidians, corals etc that could affect these physiological processes. The anti-fouling Tirol compounds (callytriols A-E) derived from the marine sponge *Callyspongia truncata* showed potent metamorphosis inducing activity against larval settlement of the ascidian *Halozynthia roretzi* at ED50 values of 0.24–4.5 μg/mL.^24^ Further, Haliclonacyclamine and halaminol A were isolated from other haplosclerid sponges and inhibited the larval settlement of ascidians *C. intestinalis* and *Herdmania momus* at concentrations as low as 5 µg/mL. Both compounds significantly induced metamorphosis and changed larval morphologies after reaching the tail-resorbed stage, and inhibited larval settlement.^25^

Some marine natural products isolated from ascidian species themselves reveal promising in-vitro activity such as anti-bacterial, anti-fungal, anti-viral, anti-malarial and anti-cancer activity.^26^ Therefore, an obvious question is whether any compounds from biofoulers such as ascidians could be found and developed for use as antifouling agents. Crude extracts from the Mediterranean ascidian *Polysyncraton lacazei* exhibited good protection against the epibiosis of *Loxocalyx* sp on the colony surface and inhibited anti-mitotic activity in sea urchin eggs at concentration 8 ppm.^27^ Other marine alkaloid compounds, Eudistomis G and H were isolated from the colonial ascidian *Eudistoma olivaceum* collected in the Indian River lagoon, United States.^28,29^ Both compounds effectively showed anti-fouling activity against the larvae of the bryozoan *Bugula neritina* at concentration 0.5%. The ascidian *E. olivaceum* showed strong anti-fouling activity against larval settlement of *B. neritina* compared to *E. capsulatum*.^30^ In addition, Murugan and Ramasamy^31^ reported modest anti-fouling activity of crude compounds from the Indian ascidian *Distaplia nathensis* against byssal thread production and attachment in the mussel *Perna indica* with EC_50_ and LC_50_ values of (50, 150 μg/mL). In further research, Trepos et al.^32^ reported novel anti-fouling compounds Synoxazolidinones A and C (**1**), Pulmonarins A and B from the sub-Artic ascidian *Synoicum pulmonaria* and screened them against sixteen bacterial and micro algae strains. Synoxazolidinone A showed potential anti-bacterial inhibition against the marine bacterial strain *Halomonas aquamarina* at a concentration of 20 µM. Synoxazolidinone A also inhibited the settlement of the microalga *Cylindrotheca closterium* and reduced the settlement of barnacle larvae at a concentration of 15 µM, which is lower than the anti-fouling activity of marine sponge *Agelas* sp. and higher than the sponge derived compound bastadin-9 and iantelline at 1 µM.^33,34^ Remarkably, a bicyclic derivative Synoxazolidinone C showed promising growth inhibition of *Balanus improvisus* at a concentration of 2 µM, which is a higher inhibition value comparing to the commercial anti-fouling product Sea Nine-211. Furthermore, Pulmonarins A exhibited strong anti-bacterial activity against *Vibrio natriegens*, and *Roseobacter littoralis* at an outstanding concentration of 30 nM, but no significant activity against micro algal species was evidenced.^34^ From this literature survey, it is clear that ascidians contain a vast pool of anti-fouling resources. Further exploration of novel biomolecules might provide promising candidates to control the benthic marine biofouling community.

### Chemical ecology of ascidians

Marine ascidians contain a broad range of natural products that can serve as important source of new therapeutics but also chemical defenses.^26^ Metabolomics provides a snapshot of the metabolites present in a living organism. It can be used in: functional genomics to analyze fluxes in metabolic pathways and to decipher the biological relevance of each metabolite, to differentiate inter and intra specific variation of marine organisms, in chemotaxonomic classification, and also in understanding the interactions of organisms with their environment.^35^ Modern developments in analytical methods have resulted in many different platforms of metabolome investigation of marine flora and fauna using mass spectrometry (MS) and Nuclear Magnetic Resonance (NMR) combined with multivariate statistical analysis.^26^ In recent years, LC-MS and NMR-based metabolomics are increasingly utilized for their systematic manner of profiling chemical fingerprints of individual samples, either plant or animals.^36,37,38^ A comparative metabolomics profiling requires a large number of samples to generate results that are statistically robust. Besides, highly sensitive and accurate instrumentation, powerful bioinformatic tools (e.g. XCMS-METLIN) are essential to address the vast amount of data generated by these experiments.^39^

Tianero and co-workers reported that microbiomes and metabolomes of ascidians contain species-specific and location- specific components.^40^ To assess the chemical diversity of ascidians, the authors applied an untargeted LC-MS based metabolomics approach. High amounts of lipids (phospho-glycerolipids, glycerolipids) were detected in ascidians and specimens were discriminated *via* geographical location rather than by species. These observations might be due to the variation of surface water temperature, available food sources and biogeographical affinity of species. Species-specific bacteria were represented by abundant OTU of the microbiomes sampled and these major components of the microbiome may be responsible for the production of the secondary metabolites.^40^ The results of this study^40^ confirmed that bacterial associations with didemnid ascidians may be related to the production of defensive chemicals in ascidians, implying a strong selection for specific bacteria. Metabolomic profiling of two Mediterranean ascidians *Styela plicata* and *Ascidia mentula* using mass spectra and multivariate analysis suggested that secondary metabolites could apparently account for chemical defence in *S. plicata*.^41^ They also recommended that the LC–MS based metabolomics method could be used as a reliable tool for taxonomic classification of marine ascidian species. Therefore, we have started some investigations into the chemical ecology of the invasive species found in Ireland, using the metabolomics approaches with the aim to find compounds of interest for future development.

In conclusion, we first report the occurrence of non- indigenous ascidians in shellfish farms in Killary Fjord, West coast of Ireland. The potential impact of NIA on the wider Irish shellfish industry needs further investigation. It is important to introduce long-term monitoring of the population of invasive ascidians *A. aspersa* and *C. eumoyata* in a longer term to determine if they persist or increase in numbers during this period. Investigation of NIA effects on blue mussel growth rate, chemical defense and other interaction in the marine environment is also recommended. In further research, symbiotic relationship with microbial communities, and MS/NMR based metabolomic profiling of these ascidians could enable straightforward detection of defense metabolites. With the high potential of secondary metabolites for the development of products that prevent marine benthic biofouling community, we believe that biodiscovery research on invasive ascidian species will have bright future.

## References

[1] Heath MR, Neat FC, Pinnegar JK, Reid DG, Sims DW, Wright P Review of climate change impacts on marine fish and shellfish around the UK and Ireland. , 2012;22(3): 337–67. doi:10.1002/aqc.2244.

[2] Davis MH, Davis ME The impact of the ascidian *Styela clava* Herdman on shellfish farming in the Bassin de Thau, France. J Applied Ichth. 2010;26(s2):12–18. doi:10.1111/j.1439-0426.2010.01496.x.

[3] Shenkar N, Swalla BJ Global diversity of Ascidiacea. PLoS One. 2011;6(6):e20657. doi:10.1371/journal.pone.0020657. PMID:21701684. 21701684 PMC3119061

[4] Minchin D Rapid coastal survey for targeted alien species associated with floating pontoons in Ireland. Aqua Invas. 2007;2(1):63–70. doi:10.3391/ai.2007.2.1.8.

[5] Marshall DJ, Pechenik JA, Keough MJ Larval activity levels and delayed metamorphosis affect post-larval performance in the colonial ascidian *Diplosoma listerianum*. Mar Eco Pro Ser. 2003;246:153–62. doi:10.3354/meps246153.

[6] Coutts ADM, Dodgshun TJ The nature and extent of organisms in vessel sea-chests: a protected mechanism for marine bioinvasions. Mar Pol Bull. 2007;54:875–86. doi:10.1016/j.marpolbul.2007.03.011. 17498747

[7] Minchin D, Nunn J, Picton B The most northern records of the exotic ascidian *Perophora japonica* Oka, 1927 (Ascidiacea: Perophoridae) in the north-east Atlantic. BioInvasions Record. 2016;5(3):139–142.

[8] Sawada H, Yokosawa H The biology of ascidians. Lambert C. C. (Ed.). Tokyo: Springer 2001.

[9] Lynch SA, Darmody G, O'Dwyer K, Gallagher MC, Nolan S, McAllen R, Culloty SC Biology of the invasive ascidian *Ascidiella aspersa* in its native habitat: Reproductive patterns and parasite load. Estu Coast Shelf Sci. 206;181:249–55. doi:10.1016/j.ecss.2016.08.048.

[10] Lambert G The south temperate and Antarctic ascidian *Corella eumyota* reported in two harbours in north-western France. J Mar Biol Asso UK. 2004;84(01):239–41. doi:10.1017/S0025315404009105h.

[11] Lynch SA, Darmody G, O'Dwyer K, Gallagher MC, Nolan S, McAllen R, Culloty SC Biology of the invasive ascidian *Ascidiella aspersa* in its native habitat: Reproductive patterns and parasite load. Estu Coastal Shelf Sci. 2016;181:249–55. doi:10.1016/j.ecss.2016.08.048.

[12] Ojaveer H, Galil BS, Minchin D, Olenin S, Amorim A, Canning-Clode J, Chainho P, Copp GH, Gollasch S, Jelmert A, Lehtiniemi M Ten recommendations for advancing the assessment and management of non-indigenous species in marine ecosystems. Mar Policy. 2014;44:160–65. doi:10.1016/j.marpol.2013.08.019.

[13] Roje-Busatto R, Ujević I PSP toxins profile in ascidian *Microcosmus vulgaris* (Heller, 1877) after human poisoning in Croatia (Adriatic Sea). Toxicon. 2014, 79:28–36. doi:10.1016/j.toxicon.2013.12.014. PMID:24418175. 24418175

[14] Minchin D, Davis MH, Davis ME Spread of the Asian tunicate *Styela clava* Herdman, 1882 to the east and south-west coasts of Ireland. Aquatic Invasions. 2006;1(2):91–6. doi:10.3391/ai.2006.1.2.7.

[15] Ardura A, Juanes F, Planes S, Garcia-Vazquez E Rate of biological invasions is lower in coastal marine protected areas. Scientific Reports. 2016;6:33013. doi:10.1038/srep33013. PMC5016778 27609423

[16] Fletcher LM, Forrest BM, Bell JJ Impacts of the invasive ascidian *Didemnum vexillum* on green-lipped mussel *Perna canaliculus* aquaculture in New Zealand. Aqua Env Inter. 2013;4(1):17–30. doi:10.3354/aei00069.

[17] Darbyson EA, Hanson JM, Locke A, Willison JHM (2009) Settlement and potential for transport of clubbed tunicate (*Styela clava*) on boat hulls. Aqu Inva 4:95–103. doi:10.3391/ai.2009.4.1.10.

[18] Bellas J Comparative toxicity of alternative antifouling biocides on embryos and larvae of marine invertebrates. Sci Total Env. 2006;367(2):573–85. doi:10.1016/j.scitotenv.2006.01.028. 16545431

[19] Bellas J Toxicity assessment of the antifouling compound zinc pyrithione using early developmental stages of the ascidian *Ciona intestinalis*. Biofouling. 2005;21(5–6), 289–96. doi:10.1080/08927010500456589. PMID:16522542. 16522542

[20] Cima F, Bragadin M, Ballarin L Toxic effects of new antifouling compounds on tunicate haemocytes: I. Sea-Nine 211™ and chlorothalonil. Aqua toxi. 2008;86(2):299–312. doi:10.1016/j.aquatox.2007.11.010. 18155783

[21] Menin A, Ballarin L, Bragadi n M, Cima F Immunotoxicity in ascidians: Antifouling compounds alternative to organotins–II. The case of Diuron and TCMS pyridine. J Env Sci Health Part B. 2008;43(8):644–54. doi:10.1080/03601230802352690. 18941987

[22] Willis KJ, Woods CM Managing invasive *Styela clava* populations: Inhibiting larval recruitment with medetomidine. Aqua Inva. 2011;6(4):511–14. doi:10.3391/ai.2011.6.4.16.

[23] Pawlik JR In Ecological Roles of Marine Natural Products; Paul VJ, Ed.; Cornell University Press: New York, 1992; pp 189–236.

[24] Tsukamoto S, Kato H, Hirota H, Fusetani N Seven new polyacetylene derivatives, showing both potent metamorphosis-inducing activity in ascidian larvae and antifouling activity against barnacle larvae, from the marine sponge *Callyspongia truncata*. J Nat Pro. 1997;60(2):126–130. doi:10.1021/np9606097.

[25] Charan RD, Garson MJ, Brereton IM, Willis AC, Hooper JN Haliclonacyclamines A and B, cytotoxic alkaloids from the tropical marine sponge *Haliclona* sp. Tetrahedron 1996;52(27):9111–20. doi:10.1016/0040-4020(96)00436-X.

[26] Palanisamy SK, Rajendran NM, Marino A Natural Products Diversity of Marine Ascidians (Tunicates; Ascidiacea) and Successful Drugs in Clinical Development. Nat Pro Biopro. 2017;7(1):1–111 doi:10.1007/s13659-016-0115-5. PMC5315671 28097641

[27] Wahl M, Banaigs B Marine epibiosis. III. Possible antifouling defense adaptations in *Polysyncraton lacazei* (Giard)(Didemnidae, Ascidiacea). J Exp Mar biol eco. 1991;145(1):49–63. doi:10.1016/0022-0981(91)90005-H.

[28] Davis AR, Wright AE Inhibition of larval settlement by natural products from the ascidian, *Eudistoma olivaceum* (Van Name). J Chem Eco. 1990;16(4):1349–57. doi:10.1007/BF01021031. 24263732

[29] Davis AR Alkaloids and ascidian chemical defense: Evidence for the ecological role of natural products from *Eudistoma olivaceum*. Mar Biol. 1991;111(3):375–9. doi:10.1007/BF01319409.

[30] Davis AR, Wright AE Interspecific differences in fouling of two congeneric ascidians (*Eudistoma olivaceum* and *E. capsulatum*): is surface acidity an effective defense?. Mar Biol. 1989;102(4):491–7. doi:10.1007/BF00438350.

[31] Murugan A, Ramasamy MS Biofouling deterrent activity of the natural product from ascidian, *Distaplia nathensis* Chordata.. 2003;32(2):162–64.

[32] Trepos R, Cervin G, Hellio C, Pavia H, Stensen W, Stensvåg K, Svendson JS, Haug T, Svenson J Antifouling compounds from the sub-arctic ascidian *Synoicum pulmonaria*: Synoxazolidinones A and C, pulmonarins A and B, and synthetic analogues. J nat pro. 2014;77(9):2105–13. doi:10.1021/np5005032. 25181423

[33] Ortlepp S, Sjögren M, Dahlström M, Weber H, Ebel R, Edrada R Thoms C, Schupp P, Bohlin L, Proksch P Antifouling activity of bromotyrosine-derived sponge metabolites and synthetic analogues. Marine Biotech. 2007;9(6):776–85. doi:10.1007/s10126-007-9029-x. 17713818

[34] Hanssen KO, Cervin G, Trepos R, Petitbois J, Haug T, Hansen E, Andersen JH, Pavia H, Hellio C, Svenson J The bromotyrosine derivative ianthelline isolated from the Arctic marine sponge *Stryphnus fortis* inhibits marine micro-and macrobiofouling. Marine biotech. 2014;16(6):684–94. doi:10.1007/s10126-014-9583-y. 25051957

[35] Longnecker K, Futrelle J, Coburn E, Soule MCK, Kujawinski EB Environmental metabolomics: Databases and tools for data analysis. Marine Chem. 2015;177:366–73. doi:10.1016/j.marchem.2015.06.012.

[36] Kim HK, Choi YH, Verpoorte R NMR-based plant metabolomics: where do we stand, where do we go? Trends Biotech. 2011;29(6):267–75. 10.1016/j.tibtech.2011.02.001 21435731

[37] Beckonert O, Keun HC, Ebbels TM, Bundy J, Holmes E, Lindon JC, Nicholson JK Metabolic profiling, metabolomic and metabonomic procedures for NMR spectroscopy of urine, plasma, serum and tissue extracts. Nature Protocols. 2007;2(11):2692–703. doi:10.1038/nprot.2007.376. PMID:18007604. 18007604

[38] Cachet N, Genta-Jouve G, Ivanisevic J, Chevaldonné P, Sinniger F, Culioli G, Perez T, Thomas OP Metabolomic profiling reveals deep chemical divergence between two morphotypes of the zoanthid *Parazoanthus axinellae*. Scientific Rep. 2015;5:8282. doi:10.1038/srep08282. PMC4319174 25655432

[39] Tautenhahn R, Cho K, Uritboonthai W, Zhu Z, Patti GJ, Siuzdak G An accelerated workflow for untargeted metabolomics using the METLIN database. Nature Biotech. 2012;30(9):826–8. doi:10.1038/nbt.2348. PMC3666346 22965049

[40] Tianero MDB, Kwan JC, Wyche TP, Presson AP, Koch M, Barrows LR, Bugni TS, Schmidt EW Species specificity of symbiosis and secondary metabolism in ascidians. ISME J. 2015;9(3):615–28. doi:10.1038/ismej.2014.152. PMID:25171330. 25171330 PMC4331574

[41] Palanisamy SK, Trisciuoglio D, Zwergel C, Del Bufalo D, Mai A Metabolite profiling of ascidian Styela plicata using LC–MS with multivariate statistical analysis and their antitumor activity. J Enzyme Inhi Med Chem. 2017;32(1), 614–23. doi:10.1080/14756366.2016.1266344. PMC6010017 28234548

